# Drug-therapy-related problems and pharmacist interventions in the medical ward in northeast Ethiopia: focus on types, acceptability, and impacts

**DOI:** 10.3389/fphar.2025.1558864

**Published:** 2025-04-07

**Authors:** Bedilu Linger Endalifer, Yared Dergu Ayta, Abate Wondesen Tsigie, Yehualashet Teshome Wondmkun, Mekuanint Terefe Kassa, Gedefaw Getnet Amare, Yenesew Wudu Ejigu, Manaye Tamrie Derseh, Abyou Seyfu Ambaye

**Affiliations:** ^1^ Departement of Pharmacy, Asrat Woldeyes Health Science Campus, Debre Berhan University, Debre Berhan, Ethiopia; ^2^ School of Medicine, Asrat Woldeyes Health Science Campus, Debre Berhan University, Debre Berhan, Ethiopia; ^3^ School of Pharmacy, College of Medicine and Health Science, Wollo University, Dessie, Ethiopia; ^4^ Department of Pharmacy, College of Health Science, Debre Markos University, Debre Markos, Ethiopia

**Keywords:** drug-related problem, intervention, impact, cause, acceptance

## Abstract

**Objective:**

This study aims to identify drug-therapy-related problems, possible interventions, acceptability, and impacts of the interventions among patients admitted to the medical ward.

**Methods:**

A hospital-based prospective interventional study was conducted in Hakim Gizaw Hospital, Debre Berhan City, Ethiopia. The sample size for the study was determined using a single proportion formula and 183 participants were recruited accordingly. Data were collected by two clinical pharmacists using a predesigned tool. The drug-therapy-related problems, interventions, and acceptability of the interventions were categorized on the basis of the Pharmaceutical Care Network Europe V.9.1 tool. The impacts of the interventions were then assessed using the clinical, economic, and organizational multidimensional tool. The data were analyzed using SPSS version 26 software.

**Result:**

Drug-therapy-related problems were identified in 27.3% of the patients, with an average incidence of 2.36 ± 0.76 events per patient. The treatment-effectiveness-related problems accounted for half (60/121) of these drug-related problems, followed by drug-selection-related causes (31/121, 26.3%), dose selection (19/121, 16.1%), and other undefined but related causes (19/121, 16.1%). A total of 143 interventions were delivered by the clinical pharmacists, of which those discussed with the prescriber (55, 38.4%) were the most frequent type. Approximately three-fourths (106/143) of these interventions were accepted. Among the pharmacist interventions, 36.4% had minor, 8.4% had major, and 4.9% showed negative clinical impacts. Economically speaking, 48.2% of the interventions were found to reduce treatment costs; organizationally, approximately 28.7% of the interventions had improved the quality of care while 14.0% had worsened it. The duration of hospitalization, comorbidities, and admission locations were observed to significantly influence the drug-related problems.

**Conclusion:**

There was a high prevalence of drug-therapy-related problems as well as high acceptance rate of interventions in the medical ward, which were found to have pronounced economic, clinical, and organizational impacts.

## Introduction

Pharmacotherapy is widely believed to enhance the health and wellbeing of patients, but its advantages may be undermined by issues with medication therapies (Abraham, 2013). Any undesirable event associated with medication therapy that genuinely or potentially compromises the intended course of treatment is referred to as a drug-related problem (DRP) (P. C. N. E. Association et al., 2020). DRPs can arise at any stage of the therapeutic process, but they are typically caused by pharmaceutical therapy prescriptions, transcribing, dispensing, and patient usage (Bekele et al., 2021a). Drug-related morbidities and mortality or poor treatment outcomes in hospitalized and outpatient settings are significantly increased by undiagnosed and unresolved DRPs (Bekele et al., 2021a; Niriayo et al., 2018; Mekonnen et al., 2024; Tefera et al., 2020). Addressing these issues through targeted interventions can prevent or minimize the risk factors and adverse health outcomes, offering significant benefits in terms of health economics and quality of life (Bekele et al., 2021b; Hussein, 2014).

However, rapid evolution of the healthcare landscape has resulted in new challenges, such as increasing numbers of available drugs, growing patient population, and more complex drug regimens. These complexities contribute to higher incidences of side effects and adverse drug reactions as well as the need for more intensive follow-up. Moreover, continuous introduction and availability of new medicines as well as the constant efflux of new information makes it practically impossible for healthcare professionals to remain updated in all aspects (Gizaw, 2017). Pharmacists play key roles in identifying, resolving, and preventing DRPs through evidence-based pharmaceutical practices (Bekele et al., 2021a). A systematic review of sixteen articles including patients with chronic diseases from different regions of the world reported that the drug-related morbidity and mortality costs accounted for approximately $177.4 billion (Braun et al., 2012). In the United States, admissions for long-term care secondary to DRPs accounted for nearly $32.8 billion (Ernst and Grizzle, 2001).

A prospective study conducted in a medical ward in Jordan reported that approximately 98.3% of all admitted patients had DRPs, with an average of 9.35 DRPs per patient (Gashaw et al., 2017). Another study involving 105 DRPs detected in cardiovascular patients in Ethiopia reported that the majority of patients experienced indication-related effects (Gobezie et al., 2014). According to a study conducted at a tertiary-care teaching hospital in India, the most common DRP was drug interactions (47.55%), followed by drug-use problems (19.58%) (Sarfaraz et al., 2017). In another study conducted in India, approximately 78.27% of the patients showed DRPs (Reshma et al., 2020). A study conducted in northeast Ethiopia reported that approximately 75.51% of the participants experienced at least one drug-therapy-related problem and that the most common DRPs were the need for additional drug therapy (35.85%), followed by unnecessary drug therapy (30.19%), and very low dosage administrations (13.2%) (Belayneh et al., 2018). In another study conducted in southwest Ethiopia, 331 DRPs were identified, with an average incidence of 1.06 DRPs per patient (Bekele et al., 2021b). In a study conducted in Tikur Anbessa, Addis Ababa, 42.3% of the DRPs noted were attributable to very low dosages (28.0%) and ineffective drug therapies (26.1%) (Demoz et al., 2019).

The Pharmaceutical Care Network Europe V.9.1 (PCNE v9.1) tool was selected in this study owing to its comprehensive approach toward categorizing DRPs, which is particularly beneficial in capturing the complexity of drug-therapy issues in clinical settings. Unlike other DRP classification tools, PCNE v9.1 classifies the DRPs based on domains and subdomains of possible causes and has been validated across different health institutions to provide reproducible results. In addition, it allows classification of the acceptance of interventions at different levels as well as the interventional outcomes (P. C. N. E. Association et al., 2020). The present study was conducted in a medical ward and highlights the high prevalence of DRPs, necessitating pharmacist-led interventions like dose adjustments and therapy optimization. These interventions are crucial for improving patient safety, enhancing treatment outcomes, and reducing hospital stays and healthcare costs, despite the challenges in acceptability and implementation. Most of the previous studies on DRPs focus on identifying and categorizing drug-therapy problems, but very few studies have comprehensively assessed the acceptability of interventions and their impacts on the clinical, economic, and organizational (CLEO) outcomes, particularly in Ethiopia. Given the resource-constrained healthcare setting in Ethiopia, understanding these aspects is crucial to improving patient outcomes while optimizing healthcare resources utilization. Furthermore, there are limited data on the conditions under which healthcare providers accept clinical pharmacy interventions as essential for successful pharmaceutical care. The findings of this study offer evidence-based insights for hospital administrators and policymakers, ultimately supporting the optimization of pharmaceutical care in other healthcare settings.

## Methodology

### Study area and study design

The present study was conducted in Hakim Gizaw Hospital (HGH) in Debre Berhan city located in the North Shewa Zone of Amhara Region, approximately 130 km northeast of Addis Ababa, the capital of Ethiopia. The hospital has different wards, among which the medical ward offers specialized medical services to the patients. A hospital-based prospective interventional study was conducted from January 1 to April 30, 2024. On average, approximately 31 patients were treated at a time in the ward, and their hospital stays averaged 7 d. This research was conducted as per the ethical guidelines of the Declaration of Helsinki. Before data collection, an ethical clearance and ethical approval letter were granted and obtained from the Institutional Review Board of Debre Berhan University Asrat Woldeyes Health Science Campus (IRB/01/127/2024). The study participants were informed about the purpose and significance of the study, and voluntary written consent was obtained from each participant before data collection. The rights, dignity, privacy, and confidentiality of all participants were respected throughout the study.

### Sample size and sampling technique

The sample size for the study was determined using a single proportion formula based on a previously reported DRP prevalence of 75.51% (Bekele et al., 2021b).

$$n=\frac{p*q*z^{2}}{d^{2}}.$$

$$n=0.7551*0.2249*1.\frac{96^{2}}{0.05^{2}}=285.$$

During the study period, approximately 510 patients were admitted, and this number was calculated from the admission records of the previous 4 months for the medical ward. Therefore, we used an adjusted formula because the study population is less than 10,000. Based on the adjusted formula shown below, 183 study participants were selected.

$$nf=n*\frac{N}{N+n}=285*\frac{510}{285+510}=182.8\approx 183.$$

Thus, the total expected number of patient admissions over the 4-month study period was approximately 510. Based on the sample size calculations, 183 participants were required for the present study. To achieve this, we determined the sampling interval (k) as follows:

$$K=\frac{N}{n}=K=\frac{510}{183\;}\approx 3,$$

where *N* is the total population (510) and *n* is the required sample size (183). The first participant was selected randomly from the first three admissions during the study period using a lottery method. Subsequently, every third admitted patient was included in the study until the sample size of 183 was reached.

### Inclusion and exclusion criteria

All patients who were admitted to the medical ward of HGH during the study period were included as long as they stayed for more than 24 h and received at least one drug. In this study, total parenteral nutrition, oxygen therapy, whole blood, and diagnostic agents were not considered drugs as they were not categorized as drugs. Patients were strictly followed from admission until discharge or transfer and death.

### Data collection process

**FIGURE 1:**
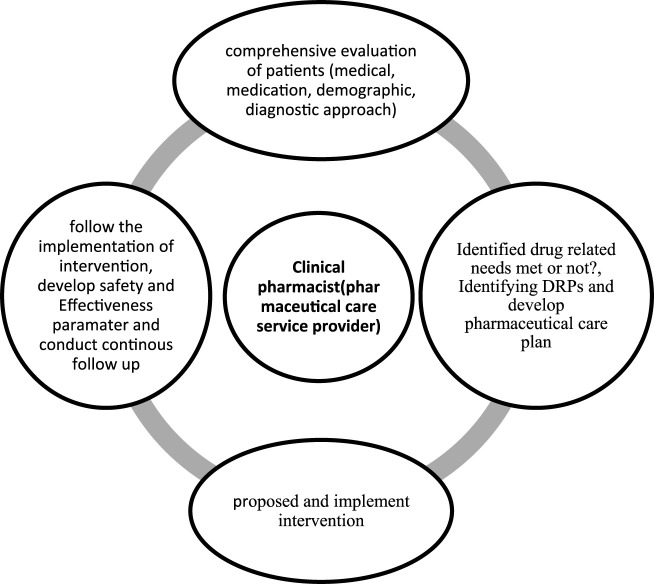
Clinical pharmacist intervention model.

The data collection tool was developed through a rigorous process of reviewing and synthesizing relevant information from previously published articles (Belayneh et al., 2018; Ahmed et al., 2024; Lekpittaya et al., 2024; Deawjaroen et al., 2022). The tool was reviewed by expert clinical pharmacists, physicians, and senior researchers to assess its clarity, completeness, and relevance in gathering pertinent information. The final tool was evaluated in 18 patients through pretests before the actual study for consistency in data collection to minimize variability. In addition, the data collection tool was evaluated using Cronbach’s alpha; our analysis yielded a Cronbach’s alpha value of 0.80, suggesting a high level of reliability. Two clinical pharmacists were responsible for data collection through regular follow-up during the working hours and on weekends, except at night, over the study period. These clinical pharmacists were selected on the basis of willingness and experience of more than 3 years in clinical pharmacy services. Once the data collection was complete, the clinical pharmacists discussed and decided upon the presence of DRPs based on the Ethiopian hospital standard treatment guidelines (Ministry of Health Ethiopia, 2021), hospital-based treatment protocols such as those for acute diabetes mellitus and acute exacerbation of asthma, and trusted sources with up-to-date information through institutional subscriptions. All included patients were followed thoroughly from admission to discharge, transfer, or death (Figure 1). The identified DRPs were discussed with the physicians, nurses, and patients at the time, and the proposed interventions were administered accordingly. The clinical pharmacists could propose more than one intervention for a single DRP based on the available evidence. In the case of disagreements between the clinical pharmacists and physicians regarding the identified DRPs and proposed interventions, discussions were held during multidisciplinary team rounds and morning sessions composed of specialist physicians, general practitioners, a nurse, and the clinical pharmacists. Finally, the identified DRPs, types of interventions, and acceptance levels of the interventions were documented with a predesigned data abstraction tool.

### DRPs, interventions, acceptance, and impacts of the interventions

The identified DRPs were categorized into three primary domains as P1: treatment effectiveness, P2: treatment safety, and P3: others, in addition to nine primary domains for causes, five primary domains for interventions, three primary domains for acceptance levels, and four primary domains for problem status according to the PCNE classification. Moreover, the more detailed levels were grouped into seven domains for problems and 43 domains for DRP causes. The planned interventions were coded as 10: no intervention, 11: at prescriber level, 12: at patient level, 13: at drug level, and 14: other types of interventions with subdomains. The intervention acceptance levels were categorized as A1: intervention accepted, A2: intervention not accepted, and A3: different types of acceptance levels (P. C. N. E. Association et al., 2020). In addition, any drug interactions were identified using an up-to-date drug interaction checker to consider only the significant interactions, specifically those classified as category X (avoid combination) and category D (consider therapy modification). The impacts of the pharmacist interventions were determined using a multidimensional tool in terms of the CLEO impacts; this tool was developed by Vo et al. (2021) as a comprehensive, validated, reliable, and feasible method for the assessment of the CLEO impacts of pharmacist interventions. The clinical impacts were assessed at six levels (−1 to 4), while the economic and organizational impacts were determined at three levels (−1 to 1) (Vo et al., 2021).

### Data analysis

The data were coded, entered, and analyzed using SPSS version 26. Descriptive statistics, including mean, median, and percentages, were calculated to present the results in tables and charts. We incorporated variables with *p*-values <0.25 from the univariate analysis in the multivariate logistic regression model. Multivariate binary logistic regression analysis was performed, and variables having *p*-values <0.05 were considered to be statistically significant. The adjusted odds ratio (AOR) and 95% confidence interval (CI) were calculated for each variable to assess the strengths of the associations.

## Results

### Sociodemographic and clinical characteristics

A total of 183 patients were included in the study, of which 97 (53.0%) were males. The average age of the patients was 44.73 ± 19.10 years, and 94 patients (51.4%) lived in urban areas. A majority of the patients were married (89, 48.6%) and were admitted from the emergency department (102, 55.7%). A large proportion of the patients (27, 14.8%) reported consuming at least one form of social drug (alcohol, chat, and tobacco). The majority of patients (162, 88.5%) were discharged with improvements, whereas only two patients (1.1%) died (Table 1).

**TABLE 1:** Sociodemographic and clinical characteristics of the study participants in HGH (n = 183).

Characteristics	Details	Frequency	Percentage (%)
Age	Mean ± SD	44.73 ± 19.10	
Sex	Male	97	53.0
	Female	86	47.0
Duration of hospitalization (days)	≤5	85	46.4
	6–10	72	39.3
	≥11	26	14.2
	Mean ± SD	5.88 ± 3.587	
Resident	Urban	94	51.4
	Rural	89	48.6
Marital status	Single	31	16.9
	Married	89	48.6
	Divorced	32	17.5
	Widowed	31	16.9
Admission location	From emergency department	102	55.7
	Referred from other institution	44	24.1
	Admission from outpatient department	37	20.2
Social drug use	Yes	27	14.8
	No	156	85.2
Comorbidities	Yes	120	65.6
	No	63	34.4
Previous hospital admission	No	136	74.3
	Once	31	16.9
	Twice or more	16	8.7
Outcome	Discharge with improvement	162	88.5
	Transferred	12	6.6
	Left against medical advice	7	3.8
	Died	2	1.1

### Disease characteristics

The most common diagnoses during admission were acute exacerbation of bronchial asthma (32, 17.5%), severe community-acquired pneumonia (30, 16.4%), and congestive heart failure (26, 14.2%) (Table 2).

**TABLE 2:** Disease characteristics of the study participants at HGH (n = 183).

Variable	Frequency	Percentage (%)
Asthma	32	17.5
Severe community-acquired pneumonia	30	16.4
Congestive heart failure	26	14.2
Diabetes mellitus	14	7.7
Sepsis	14	7.7
Pulmonary embolism and deep-vein thromboembolism	11	6.0
Chronic kidney disease/Acute kidney injury	8	4.4
Stroke	7	3.8
Hypertensive crisis	6	3.3
Malaria	5	2.7
Gastroenteritis	4	2.2
Meningitis	4	2.2
Hypo/Hyperkalemia	3	1.6
Myocardial infarction	3	1.6
Glomerulonephritis	3	1.6
Peptic ulcer	2	1.1
Anemia	2	1.1
Chronic liver disease	2	1.1
Others	7	3.8

Others: Acute hepatitis, complicated urinary tract infection, tetanus, cellulitis, rash/hypersensitivity.

### Types and numbers of DRPs

**FIGURE 2:**
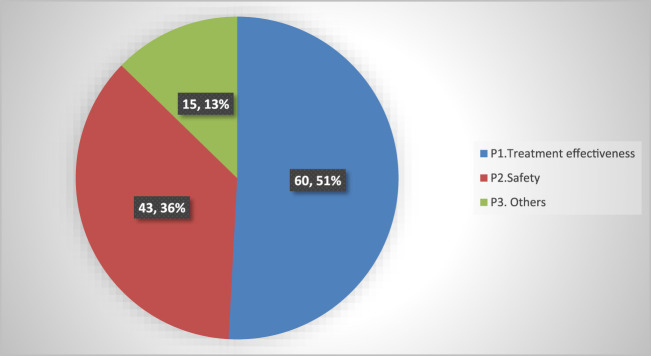
Classification of the drug-related problems (DRPs) by primary domains among the study participants in Hakim Gizaw Hospital (HGH; n = 183).

Among all the patients who participated in this study, 50 individuals (27.3%) encountered at least one drug-therapy problem, with the average value of the entire study cohort being 2.36 ± 0.76. A total of 118 DRPs were identified among the study participants. Of these, 14 patients (7.7%) had one DRP, nine patients (4.9%) had two DRPs, 22 patients (12.0%) had three DRPs, and five patients (2.7%) had a maximum of four DRPs each. The most common category of DRPs was related to treatment effectiveness, which accounted for 60 cases (51.0%) (Figure 2).

### Commonly identified causes of DRPs

Drug-selection-related causes (C1) accounted for the highest proportion of DRPs (31, 26.3%), among which inappropriate drug selection according to guidelines (C1.1) (8, 6.8%) and incomplete or no drug treatment despite existing indication (C1.5) (3, 2.5%) accounted for the highest and lowest frequency, respectively. Among the dose-selection related causes (C3), very low dose (C3.1) (7, 5.9%) had the highest frequency, while drug–drug interactions had the highest frequency (13, 11.1%) under other causes (C9.2) (Table 3).

**TABLE 3:** Common causes of DRPs in the study participants in HGH.

Primary domain	Cause	Frequency (percentage)
C1. Drug selection (31)	C1.1. Inappropriate drug as per guidelines/formulary	8 (6.8%)
	C1.2. No drug indications	6 (5.1%)
	C1.3. Inappropriate combination of drugs, drugs and herbal medications, or drugs and dietary supplements	5 (4.2%)
	C1.4. Inappropriate duplication of a therapeutic group	5 (4.2%)
	C1.5. No or incomplete drug treatment	3 (2.5%)
	C1.6. Too many different drugs/active ingredients prescribed for indication	4 (3.4%)
C2. Drug form (4)	C2.1. Inappropriate drug form/formulation	4 (3.4%)
C3. Dose selection (19)	C3.1. Drug dose too low	7 (5.9%)
	C3.2. Drug dose of a single active ingredient too high	5 (4.2%)
	C3.3. Dosage regimen not frequent enough	4 (3.4%)
	C3.4. Dosage regimen too frequent	3 (2.5%)
C4. Treatment duration (8)	C4.1. Duration of treatment too short	5 (4.2%)
	C4.2. Duration of treatment too long	3 (2.5%)
C5. Dispensing (12)	C5.1. Prescribed drugs not available	3 (2.5%)
	C5.2. Necessary information not provided	6 (5.1%)
	C5.3. Wrong drug, strength, or dosage advised (OTC)	3 (2.5%)
C6. Drug use process (14)	C6.1. Inappropriate timing of administration or dosing intervals by a health professional	5 (4.2%)
	C6.2. Drug underadministered by a health professional	3 (2.5%)
	C6.3. Drug overadministered by a health professional	2 (1.7%)
C7. Patient related	C7.2. Patient uses/takes more drugs than prescribed	2 (1.7%)
	C7.5. Patient consumes food that causes interactions	5 (4.2%)
	C7.6. Patient physically unable to use the drug/form as directed	2 (1.7%)
	C7.7. Patient unable to understand instructions properly	2 (1.7%)
C8. Patient transfer related (4)	C8.1. Medication reconciliation problem	4 (3.4%)
C9. Others (19)	C9.1. No or inappropriate outcome monitoring (including TDM)	6 (5.1%)
	C9.2. Drug–drug interaction (category X = 5, category D = 8)	13 (11.1%)

### Planned interventions

The clinical pharmacists delivered 143 interventions to resolve the 118 identified DRPs. Among the prescriber-level interventions (11), those discussed with the prescriber (55, 38.4%) were the most frequent type. Among the drug-level interventions (13), stopped or paused drugs accounted for the maximum cases (21, 14.7%); among the patient-level interventions (12), patient counseling on drugs was the most frequent (11, 7.7%) (Table 4).

**TABLE 4:** Types of proposed interventions in the study participants in HGH.

Primary domain	Intervention	Frequency (%)
11. At the prescriber level (62)	11.2. Prescriber asked for information	4 (2.8)
	11.3. Intervention proposed to prescriber	3 (2.1)
	11.4. Intervention discussed with prescriber	55 (38.4)
12. At the patient level (17)	12.1. Patient (drug) counseling	11 (7.7)
	12.4. Spoken to family member/caregiver	6 (4.2)
13. At the drug level (60)	13.1. Drug changed	11 (7.7)
	13.2. Dosage changed	14 (9.8)
	13.3. Formulation changed	6 (4.2)
	13.5. Drug paused or stopped	21 (14.7)
	13.6. Drug started	8 (5.6)
14. Other (4)	14.1. Other intervention	4 (2.8)

### Acceptance of the intervention

A total of 143 interventions were administered to manage all 118 DRPs, so the average intervention rate was 1.21 per DRP. Of these, 106 interventions (74.1%) were completely accepted, 77 interventions (53.8%) were fully implemented, and 27 interventions (18.9%) were not accepted (Table 5).

**TABLE 5:** Acceptance of pharmacist interventions in the study participants in HGH.

Primary domain	Implementation	Frequency (%)
A1. Intervention accepted (106, 74.1%)	A1.1. Intervention accepted and fully implemented	77 (53.8)
	A1.2. Intervention accepted but partially implemented	12 (8.4)
	A1.3. Intervention accepted but not implemented	8 (5.6)
	A1.4. Intervention accepted but implementation unknown	9 (6.3)
A2. Intervention not accepted (27, 18.9%)	A2.1. Intervention not accepted: not feasible	11 (7.7)
	A2.2. Intervention not accepted: no agreement	7 (4.9)
	A2.4. Intervention not accepted: unknown reason	9 (6.3)
A3. Other (10, 7%)	A3.1. Intervention proposed: acceptance unknown	10 (7)

### CLEO impacts of the interventions

The impacts of the 143 interventions were determined based on the multidimensional tool proposed by Vo et al. (2021). Approximately 52 interventions (36.4%) had minor impacts on the clinical outcomes of the patients, and 12 interventions (8.4%) had major clinical outcomes; however, seven interventions (4.9%) had negative clinical consequences, suggesting worsened outcomes. The pharmacist interventions were assessed for the economic impacts, which showed that 69 interventions (48.2%) decreased the treatment costs. Approximately 41 interventions (28.7%) improved the organizational quality of care, whereas 20 interventions (14.0%) hurt the organization’s quality of care (Table 6).

**TABLE 6:** Impacts of the interventions at the clinical, economic, and organizational levels in the study participants at HGH (n = 183).

Score	Impact	Frequency (%)
Clinical impact		
-1C	Negative	7 (4.9)
0C	Null	5 (3.5)
1C	Minor	52 (36.3)
2C	Moderate	30 (21.0)
3C	Major	12 (8.4)
4C	Avoid fatality	2 (1.4)
UND	Undetermined	35 (24.5)
Economic impact		
-1C	Increased cost	34 (23.8)
0C	No change	14 (9.8)
1C	Decreased cost	69 (48.2)
UND	Undetermined	26 (18.2)
Organizational impact		
-1O	Negative	20 (14.0)
0O	Null	45 (31.4)
1O	Positive	41 (28.7)
UND	Undetermined	37 (25.9)

### Factors associated with DRPs

The univariate binary logistic regression results revealed that the DRPs were significantly associated with the duration of hospitalization, comorbidities, admission locations, and previous hospitalization. In the multivariate analysis, the duration of hospitalization, comorbidities, and admission location remained significantly associated with DRPs. Accordingly, patients who stayed ≥11 d were 1.45 times more likely to experience DRPs than those who stayed ≤5 d (AOR = 1.45, 95% CI = 1.92–4.10). Compared to patients who had no comorbidities, those who had comorbidities had a two-fold increased likelihood of developing DRPs (AOR = 2.16, 95% CI = 1.27–3.70). Based on the patient’s admission location, those who were referred and admitted from the emergency department were 2.82 times more likely to experience DRPs than those admitted through the outpatient department (Table 7).

**TABLE 7:** Univariate and multivariate logistic regression results of factors associated with DRPs in the medical ward in HGH (n = 183).

Variables		DRPs		COR (95%; CI)	AOR (95%; CI)
		Yes	No		
Sex	Male	23	74	0.68 (0.35, 1.30)	
	Female	27	59	Ref.	
Duration of hospitalization	≤5	16	69	Ref.	
	6–10	21	51	1.78 (0.85–3.74)	2.53 (0.23–5.07)
	≥11	13	13	4.31 (1.68–11.00)	1.45 (1.92–4.10)*
Comorbidities	Yes	29	91	0.64 (0.43–0.95)	2.16 (1.27–3.70)*
	No	21	42	Ref.	
Admission location	From emergency department	27	75	1.86 (1.05–3.31)	2.82 (1.41–5.65)*
	Referred from other institution	17	27	3.24 (1.74–6.06)	1.54 (0.11–3.82)
	Admission from outpatient department	6	31	Ref.	
Social drug use	Yes	11	16	2.06 (1.26–3.40)	
	No	39	117	Ref.	
Previous hospitalization	No	41	95	Ref.	
	Once	8	23	0.81 (0.48–1.36)	
	Twice or above	1	15	0.15 (0.045–0.50)	
Residence	Urban	22	72	0.67 (0.346–1.28)	
	Rural	28	61	Ref.	
Marital status	Single	9	22	1.00 (1.00–1.00)	
	Married	23	66	0.85 (0.50–1.45)	
	Divorced	9	23	0.95 (0.52–1.85)	
	Widowed	9	22	Ref.	

*Significant association.

## Discussion

The DRP occurrences of hospitalized patients may be linked to various causes and risk factors. Identifying these factors is essential for preventing and avoiding the DRPs. The current study reveals that the incidence of DRPs was 27.3% among patients admitted to the medical ward. This finding is lower than the rates reported in other hospitals, which range from 52% to 96.1% in Ethiopia (Bekele et al., 2021b; Hussein, 2014; Gobezie et al., 2015; Tigabu et al., 2014), 81% in Norway (Blix et al., 2004), (57.4%) in Bangkok (Thailand) (Pramotesiri et al., 2024), 77% in China (Liu et al., 2021), 77% in Nepal (Thapa et al., 2024), and 90.5% in Malaysia (Abdulmalik et al., 2019). Our finding is also higher than the 21.0% incidence in Germany (Sell and Schaefer, 2020). These differences in the DRP rates may be explained by differences in the study design, setting sample sizes, and number of study centers. First, the data collection, identification, and interpretation of DRPs were conducted by clinical pharmacists with a minimum of 3 years of experience in hospital-based clinical pharmacy services. All pharmacists involved in our study had been trained in clinical pharmacy and were actively engaged in ward-based patient care, working closely with physicians and other healthcare professionals to optimize medication therapy. Second, as an interventional study, the present work involved continuous monitoring over a specified period that allowed the healthcare team to identify and resolve potential DRPs early, preventing their recurrence (temporal effects of continuous monitoring). Third, our study center was a new healthcare institution with a possibly lower rate of admission, which could have resulted in a higher quality of care.

The average rate of DRPs found in the present study is comparable to that of another study conducted in Thailand, which reported 1–3 DRPs per patient (Deawjaroen et al., 2022); however, the present value is higher than that reported for a study conducted in Gondar (Ethiopia) that showed an average of 1.04 DRPs per patient, with 67.4% of the subjects showing one DRP, 24.5% showing two DRPs, and 8.2% showing three DRPs (Gashaw et al., 2017). The incidence rate of DRPs in the present study is also higher than that reported for a study conducted in Dessie (Ethiopia), where the average value was 1.08 DRPs per patient (Belayneh et al., 2018). Another study conducted in Addis Ababa, Ethiopia, showed that 70.1% of the patients had one, 26.5% had two, and 3.4% had more than two DRPs (Mekonnen et al., 2024). This may be because of more clinical pharmacists participating in ward-based activities and variations in the DRP classifications. In addition, the current study focused on drug adherence problems in admitted patients, which are minimal compared to those of ambulatory patients, and organizational differences; the hospital in this study is a general facility, whereas those in the above studies were either comprehensive or specialized hospitals.

In the current study, the DRPs related to treatment effectiveness accounted for the most incidents, followed by safety, and finally those from other causes. This is in line with the findings of studies conducted in Nepal (Thapa et al., 2024) and China (Zhu et al., 2019), where treatment effectiveness and treatment safety were the major types of DRPs. Among the primary domains of DRPs, drug selection was the most prevalent cause. Within the drug selection subgroup, inappropriate drug use against the guidelines/formulary was a significant contributor, followed by drugs with no indications, which accounted for the largest proportion. Additionally, drug–drug interactions were a major factor contributing to the overall causes of DRPs in the current study. In other studies conducted in Ethiopia, additional drug therapy (Reshma et al., 2020; Gobezie et al., 2015) and inappropriate dosage (Gashaw et al., 2017) were the most common causes of DRPs, while inappropriate drug use against the guidelines or formulary were the most common causes in Nepal (Thapa et al., 2024).

In the current study, clinical pharmacists delivered the interventions for all DRPs. These proposed interventions may be attributable to full-time follow-up of patients by the pharmacists. Our study revealed that interventions at the prescriber and drug levels constituted the primary domain, while interventions discussed with the prescriber (dosage changes as subdomain) accounted for the largest portion. This finding is consistent with other reports from China, where drug-level primary-domain interventions were observed in 44.1% of cases and dosage changes were the major subdomain at 35.5% (Zhu et al., 2019); the most frequent intervention at the prescriber level was proposed to the prescribers (Deawjaroen et al., 2022). However, our findings are inconsistent with those reported from Nigeria at the prescriber level (16.99%) and patient/carer level (46.00%) (Adibe et al., 2017) as well as from India at the drug discontinuation (29.58%) and dosage change (22.53%) levels (Bramhini et al., 2022).

Based on the current findings, there is a high rate of acceptance of the pharmacist interventions, and this acceptance rate is close to those observed in previous studies in the Netherlands (71.2%) (Zaal et al., 2020), France (77.9%) (Durand et al., 2022), and southwestern Saudi Arabia over 2 study years (82.5% in the first and 70.3% in the second years) (Babelghaith et al., 2020). We found statistically significant relationships in our study between the presence of DRPs and duration of hospitalization as well as presence of comorbidities, which are in line with the findings of most previous studies (Gashaw et al., 2017; Thapa et al., 2024). Similarly, patients admitted through referrals from other health institutions were associated with a risk of DRPs. These findings may be attributed to those patients who may have had more comorbidities and complicated diseases that required critical follow-ups.

In our study, a small percentage of the pharmacist interventions were considered to have prevented potentially lethal effects, while more than one-third of the interventions had minor to major clinically significant effects. In other studies, it has been reported that 95% of such interventions have had extremely clinically significant to somewhat significant impacts (Han et al., 2016). Another study also reported that 75% of the interventions had minor to major clinical impacts (Novais et al., 2021). In our study, a limited number of pharmacist interventions had negative or neutral clinical impacts, leading to deterioration of patient condition, reduced adherence, and lower satisfaction, or no noticeable effects on patient clinical outcomes. It is known that all interventions may have positive clinical impacts, as supported by previous reports by Han et al. (2016) and Novais et al. (2021), who noted no clinically significant impact rates of 5.5% and 21.2%, respectively. A portion of these interventions increased the cost of treatment, while a smaller portion had no impact on the treatment cost. Conversely, some interventions led to decreases in treatment costs, while the cost impacts of some other interventions remained undefined. Another study from France also reported that 16.6% of the interventions increased treatment costs, while 23.8% had no impacts on cost, 55.2% decreased the cost of treatment, and 4.4% had undetermined cost impacts (Novais et al., 2021). Approximately 14.0% of interventions increased the complexity or organizational flow of care, while 31.4% did not impact the healthcare delivery process. The organizational process of healthcare delivery increased in 28.7% of the interventions, while the impact on quality of care was not determined in 25.9% of the interventions. Comparisons of the CLEO impacts of the interventions between the present study and other studies is difficult because of differences in the methodologies used, which include the numbers, professions, and educational levels of the data collectors and assessors as well as study settings, tools used, DRP classifications, and intervention categories.

The findings of this study underscore the pivotal roles of clinical pharmacists in addressing DRPs. The incidence rate of DRPs in the present study highlight the urgent need for targeted interventions to optimize drug therapy, minimize adverse drug events, and enhance patient safety in hospital settings. The acceptability of the interventions among healthcare providers suggests that collaborative approaches involving integration of pharmacists into multidisciplinary healthcare teams can improve medication management and adherence to clinical guidelines. Furthermore, our findings contribute to the effectiveness of pharmacist-led interventions, particularly in resource-limited settings like Ethiopia, where healthcare resources may be constrained. The strengths of the present study include the use of standardized PCNE V.9.1 DRP intervention and acceptance classification criteria, prospective nature, and involvement of clinical pharmacists during data collection, DRP identification, and intervention. In addition, the current study provides assessments of the CLEO impacts of pharmacist interventions, which can be used to expand the roles of clinical pharmacists in pharmaceutical care. To the best of our knowledge, the present study is a pioneering effort at describing the impacts of pharmacist interventions using the CLEO three-dimensional tool in Ethiopian hospitals. The main limitations of this study include the single-center setup, lack of 24-h coverage of the services, and inherent subjectivity in the identification and classification of pharmacist interventions. Another limitation of the present study is that it lacks financial estimations of the economic impacts of the interventions, which is an avenue for future exploration.

## Conclusion

In this study, we identified a high prevalence of DRPs in the medical ward of the HGH in Debre Berhan city, Ethiopia, that were primarily related to treatment effectiveness, including inappropriate drug selection and drugs without indications. The clinical pharmacists at the hospital intervened in all cases, and the acceptance rate for these interventions was 71.1%. The interventions had significant CLEO impacts on healthcare delivery. The length of patient stay at the hospital, referral admissions, and comorbidities were the key risk factors for the DRPs. The presence of clinical pharmacists is crucial for addressing drug-related needs as well as preventing, identifying, and resolving the DRPs, which can ultimately improve the quality of care.

## Data Availability

The original contributions presented in this study are included in the article/supplementary material, and any further inquiries may be directed to the corresponding author.

## Abbreviations

CLEO: clinical, economic, and organizational; DRP: drug-related problem; PCNE: Pharmaceutical Care Network Europe.
